# Plasma levels of leucocyte elastase-generated cross linked fibrin degradation products (E-XDP) are elevated in chronic venous disease

**DOI:** 10.1371/journal.pone.0261073

**Published:** 2021-12-14

**Authors:** Helen Sinabulya, Angela Silveira, Lena Blomgren, Joy Roy

**Affiliations:** 1 Department of Molecular Medicine and Surgery, Karolinska Institutet, Stockholm, Sweden; 2 Department of Medicine Solna, Karolinska Institutet and Karolinska University Hospital, Stockholm, Sweden; 3 Department of Cardiovascular Surgery, Karlskoga Vein Centre, Faculty of Medicine and Health, Örebro University Hospital, Örebro, Sweden; Fiji National University, FIJI

## Abstract

Patients with chronic venous disease (CVD) have elevated levels of leucocyte elastase (LE) released from the activation of leucocytes. In acute deep venous thrombosis (DVT), LE can degrade fibrin from the thrombus resulting in cross-linked fibrin degradation products (E-XDP) being released into the bloodstream. In patients with CVD the levels and significance of circulating E-XDP are unknown. We aimed to investigate the association between plasma E-XDP concentration and severity of CVD. Levels of E-XDP were quantified with a specific enzyme-linked immunosorbent assay (ELISA) in plasma from 142 consecutively recruited CVD patients (mean age 64 years, (range 23–89), 81 were females and 61 males). Patients were also divided into three groups based on CVD severity using the C-class of the Clinical-Etiological-Anatomical-Pathophysiological (CEAP) classification, with C 0–1 class as the reference group, C 2–3 as the second group and C 4–6 as the third group with the most severely affected patients. We found significantly elevated levels of E-XDP in patients with C 4–6 compared with patients with C 0–1 (p = 0.007) and increased with increasing disease severity across the groups (p = 0.02). Significant independent association was observed between levels of E-XDP and the classes C 4–6 after adjustment for age and sex (p < 0.05), but the association was no longer significant after further adjustment for use of statins, use of anticoagulants and history of DVT (p = 0.247). This exploratory study shows that E-XDP levels are elevated in patients with CVD, encouraging further studies on the role of E-XDP in CVD.

## Introduction

Chronic venous disease (CVD) is one of the most common pathologies in the adult western world population [1, 2]. The clinical manifestations of the disease vary from asymptomatic clinical findings like telangiectasies, varicose veins with or without symptoms, to more chronic and symptomatic findings like skin changes and venous leg ulcers, the latter referred to as chronic venous insufficiency (CVI) [3]. The most severe form of CVD, venous ulceration, has a prevalence of about 1% [2]. Clinical manifestations of CVD are commonly assessed by the CEAP-classification (Clinical-Etiology-Anatomy-Pathophysiology), where the C-class grades clinical signs as follows: C0 is no sign of CVD, C1 signs of telangiectasias, C2 presence of varicose veins, C3 presence of oedema, C4 hypostatic skin changes such as hyperpigmentation, eczema, lipodermatosclerosis or corona phlebectatica, C5 healed ulcer and C6 active ulcer [4].

The most common cause of CVD is varicose veins, less common post-thrombotic changes, and rarely venous malformations [5, 6]. A history of deep venous thrombosis (DVT) is commonly associated with worse clinical severity and quality of life in CVD patients [7]. Venous ulcers may also occur secondary to varicose veins. However, it is still unclear why some individuals with venous insufficiency develop skin changes, thus making the clinical diagnosis more complex as it is difficult to predict which patients are at a higher risk of developing venous ulceration. Implementation of solid disease markers could positively contribute to CVD management.

It has been shown that the development of leg ulcers involves both the activation of leucocytes that damage the tissues and skin in the leg as well as coagulopathy including thrombophilia [8–10]. Patients with venous thromboembolism have an increased level of circulating thrombolysis-related markers and the clinically used marker is D-dimer [11]. Levels of D-dimer can be affected by several factors and statin use has been suggested to decrease its levels in plasma [12]. D-dimer is a product of fibrinolysis and is included in the pool of fragments generated by proteolytic digestion of fibrin by plasmin referred to as cross-linked fibrin degradation products (XDP) [13].

Patients with CVD have elevated levels of leucocyte elastase (LE) that is released upon activation of leucocytes [14]. This enzyme degrades cross-linked fibrin specifically, in sites different from those hydrolyzed by plasmin, producing fibrin degradation products known as E-XDP which are structurally different from D-dimers [15, 16]. Circulating levels of E-XDP have previously been observed to be elevated in patients with DVT and abdominal aortic aneurysms with large intraluminal thrombi [17, 18], however to our knowledge no such study has been performed on CVD patients to date.

The aim of our study was therefore to investigate the association between plasma concentration of E-XDP in patients with various degrees of CVD severity.

## Methods

### Patients

Consecutive patients attending a high-volume centre dedicated to treatment of varicose veins in Stockholm, Sweden, were asked to participate in the study between January 2015 and April 2016. Patients that failed to understand the given information for reasons such as cognitive impairment or language difficulties were not included. A total of 142 patients accepted and were included in the study. History of DVT and if on any current medication with a statin or anticoagulant (Warfarin or any antithrombotic medication) was recorded. Clinical status was classified and graded according to the C-class of CEAP which was used to group the patients for analysis, generating 3 groups with increasing CVD severity. The reference group consisted of patients classified as C 0–1 and no history of DVT, patients classified as C 2–3 made the second group and patients classified as C 4–6 made the third group. Duplex ultrasound was used to confirm the diagnosis in all cases.

Samples of peripheral blood were drawn preceding surgical treatment or sclerotherapy. The blood samples were anti-coagulated with citrate and first centrifuged for 20 minutes at 2500 g followed by 30 minutes at 20 000 g to obtain platelet free plasma and then stored at −80°C until analysis.

### Enzyme-linked immunosorbent assay

In the plasma samples, E-XDP was measured with a sandwich enzyme-linked immunosorbent assay (ELISA) essentially as described by Kohno et al. [16]. The monoclonal antibody anti-E-XDP clone IF-123 (Cosmo Bio Co. Ltd., Tokyo, Japan) was used for catchment of E-XDP in the samples as it specifically recognizes elastase-digests of human fibrinogen and fibrin, but not their plasmin-digests. Horseradish peroxidase-labelled rabbit polyclonal antibody against human fibrinogen (DAKO, Glostrup, Denmark) was used as probing antibody and 3,3′,5,5′-tetramethylbenzidine as substrate [19]. The assay was calibrated with the Coagulation Reference Plasma from Technoclone GmbH (Vienna, Austria) defined as having 1 U E-XDP/mL. Inter-assay coefficient of variation of the assay was >10%.

### Statistical analysis

All statistical analyses were performed using SPSS version 24 (SPSS Inc. Chicago, IL, USA). The binary logarithm of E-XDP concentration was used in all analyses. Groups were treated as categorical variables. Differences of means between two groups were examined for statistical significance using the Student’s *t*-test for independent samples. Differences of more than two means between groups were calculated by the ANOVA test. To study the association between plasma E-XDP concentrations and severity of CVD, multiple linear regression with an extended model approach for the covariates was used to adjust for potential confounders. Model 1 adjusted for age and sex. Model 2 adjusted for age, sex, and other risk factors (history of DVT, use of Statin, and use of anticoagulant). We used the *p*-value 0.05 threshold to denote a statistically significant difference.

### Ethics

The Regional Ethics Review Board in Stockholm approved the study protocol registered under number 2014/3:3. All patients received written information and signed a consent form.

## Results

### Patients

Patient demographics and characteristics are shown in Table 1. A total of 142 patients were included, mean age 64 years, (range 23–89), 81 females and 61 males. Compared to the group C0-1, history of DVT and anticoagulant use were significantly higher in the groups C2-3 and C4-6. In addition, 17% of the patients in group C4-6 reported use of statins, which was not seen in the other groups.

**Table 1:** Patient characteristics and demographics.

	C0-1 (n = 10)	C2-3 (n = 41)	C4-6 (n = 91)	p-value
**Age (years), mean (SD)**	58 (14)	61 (14)	66 (13)	< 0.001^α^
**Sex, n (%)**				0.708^τ^
**Female**	9 (90)	27 (66)	45 (49)	
**Male**	1 (10)	14 (34)	46 (51)	
**History of DVT n (%)**	0	1 (2)	9 (10)	0.015^τ^
**Use of Statin n (%)**	0	0	15 (17)	0.001^τ^
**Use of anticoagulant n (%)**	0	6 (15)	25 (28)	0.001^τ^

C, C-class of CEAP classification.

SD, Standard deviation.

DVT, deep vein thrombosis.

^α^One-way ANOVA.

^τ^Student’s *t*-test.

### Plasma E-XDP concentration and chronic venous disease

**Fig 1:**
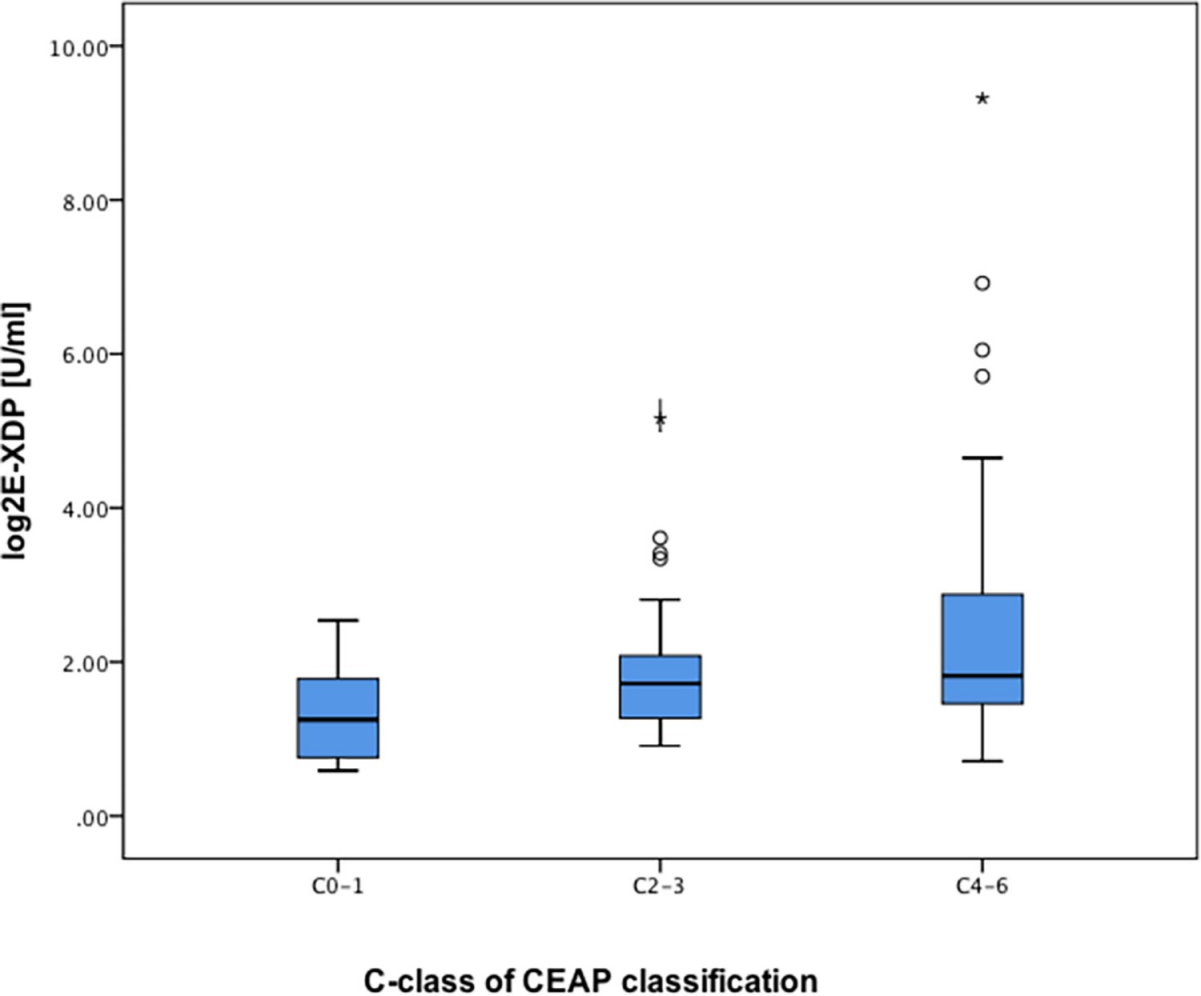
Concentration of E-XDP between groups of chronic venous disease. In the box plots, the boundary of the box closest to zero indicates the 25^th^ percentile, a black line within the box marks the median, and the boundary of the box farthest from zero indicates the 75^th^ percentile. Whiskers above and below the box indicate the 10^th^ and 90^th^ percentiles. Points above the whiskers indicate outliers outside the 90^th^ percentile.

Patients with C 4–6 had a significantly higher concentration of E-XDP compared to patients with C 0–1 (p < 0.05) (Fig 1). The following clinical features were analysed using univariate analysis, age (p < 0.001), sex (p = 0.708), use of statins (p < 0.001), use of anticoagulants (p < 0.001) and history of DVT (p = 0.015) before subjection to multiple linear regression. The analysis of residuals confirmed the assumptions of linearity.

Results of the multiple linear regression in Table 2 show that E-XDP was independently associated with clinical classes C 4–6 (p < 0.05) after adjustment for age and sex, and that further adjustment for differences significant in univariate analysis (use of statins, use of anticoagulants and history of DVT) blunted the association between higher concentrations of E-XDP and clinical classes C 4–6.

**Table 2:** Linear regression coefficients.

	C0-1 (95% CI) [n = 10]	C2-3 (95% CI) [n = 41]	C4-6 (95% CI) [n = 91]	p-value Trend
**Univariate**	Ref	0.54 (-0.32–1.40)	0.97 (0.16–1.78)	0.007
**Model 1**	Ref	0.50 (-0.33–1.34)	0.86 (0.05–1.66)	0.019
**Model 2**	Ref	0.42 (-0.37–1.22)	0.52 (-0.26–1.30)	0.247

C, C-class of CEAP classification; CI, confidence interval.

Model 1 adjusted for age and sex.

Model 2 adjusted for age, sex, and other risk factors (history of DVT, use of Statin, and use of anticoagulant).

## Discussion

Patients with chronic venous disease, CEAP clinical class C 4–6 have elevated levels of circulating E-XDP compared to patients C 0–1. The association between E-XDP and disease severity persisted after adjustment for age and sex but we have no evidence to conclude that such an association exists after adjusting for use of statins, anticoagulants and history of DVT.

As varicose veins are common, and as the advanced stages of CVD are painful for patients and costly for society, a marker for the risk of developing these stages would be welcome to better select patients for treatment in earlier stages of the disease. In many diseases such as DVT and pulmonary embolism D-dimer is elevated and its quantification is widely used clinically to assess the disease status [20, 21]. However, D-dimer lacks specificity in cases of venous thromboembolism and especially in elderly patients [22]. Because leucocyte activation with consequent release of elastin has been shown to participate in the pathophysiology of CVD, it would be reasonable to assume that E-XDP determination would be more specific for CVD than D-dimer. Of note, the assay used in the present study was proven specific for measuring levels of E-XDP generated from fibrin by the proteolytic action of LE (and not plasmin) by Kohno et al. and in a study done by our group [16, 18].

In the present report we observed a significant difference in E-XDP in patients grouped according to increased clinical severity of CVD but the reason for the elevated E-XDP in these patients remains unknown. Besides activation of leukocytes, it is possible that the patients have thrombi in other parts of the body or micro thrombi in the vein wall that are also degraded by LE, but other unknown sources of thrombi can not be excluded [14, 23]. For example, in a previous study by our group, we observed an association between E-XDP and presence of intraluminal thrombi in patients with abdominal aortic aneurysms [18].

A review of the literature suggested that use of lipophilic statins decreased plasma levels of D-dimer but the effect on XDP is unknown [12]. CVD is an inflammatory disease and markers of inflammation such as albumin, fibrinogen, D-dimer and leucocytes have also been observed to be associated with severity of CVD [24]. Our results suggest that E-XDP is increased in this group but in a multivariate model which included history of DVT, statin and anticoagulation treatment, the statistical significance was lost. We acknowledge that in this relatively small study, it is difficult to ascertain whether an underlying prothrombotic state or medical treatment could be the reason for higher levels of E-XDP and larger studies are required.

Our study has limitations which can be listed and addressed. The most severely affected patients in group C4-6 were significantly older than the patients in the other groups and many biochemical markers increase with age; however the relationships of E-XDP levels with severity of CVD were still statistically significant after adjustment by age (Model 1, Table 2). The reference group was relatively small (n = 10 patients with C 0–1) but was compensated with a distribution of the E-XDP values in this expected low range. Another limitation is the lack of symptom evaluation with impact on quality of life, which could alter the composition of the groups considering that symptoms of CVD do not always correlete with disease severity. On the other hand, computation of values from a survey could have strengthened the differences between the groups as venous ulceration leads to poorer quality of life. Also, and importantly, we have measured E-XDP in samples taken at one time point and we do not know which factors influence the increased E-XDP levels association with increasing disease severity. A follow-up of these patients ideally with multiple sampling and inclusion of a panel of biochemical parameters, not measured in this study, would address this question.

In summary, in this pilot study we found that increased levels of E-XDP were associated with increasing disease severity, an association that persisted after adjustment for age and sex, but could not be confirmed after further adjusting for use of statins and anticoagulants. It may be speculated that E-XDP levels increase over time with increasing disease severity, thus a longitudinal follow-up of (a larger group of) patients in early stages of the disease progressing to varying severity of CVD, and well characterized with more biochemical markers, would give valuable information about the role and value of E-XDP as a marker for disease progression and severity.

## Supporting information

S1 File Data file. (SAV)

S1 Table Univariate linear regression with E-XDP as dependent variable. ^1^For this analysis, CEAP class was treated as categorical variable. * Significant at p<0.05 level.
(DOCX)
