# Supplementary material for: Plasma levels of leucocyte elastase-generated cross linked fibrin degradation products (E-XDP) are elevated in chronic venous disease
Source: PLoS One. 2021 Dec 14;16(12):e0261073. doi: 10.1371/journal.pone.0261073 (PMC8670697; doi:10.1371/journal.pone.0261073)
Supplement: S1 Table — 1For this analysis, CEAP class was treated as categorical variable. * Significant at p<0.05 level. (DOCX) [file pone.0261073.s002.docx]

**S1 Table.** Univariate linear regression with E-XDP as dependent variable

| **Independent variable** | **Regression coefficient** | **95% Confidence interval** | | **P-value** | **R2** |
| --- | --- | --- | --- | --- | --- |
|  |  | *Lower bound* | *Upper bound* |  |  |
| Age | 0.03 | 0.01 | 0.04 | <0.001* | 0.09 |
| Male sex | -0.08 | -0.50 | 0.34 | 0.71 | 0 |
| History of DVT | 0.99 | 0.19 | 1.80 | 0.015* | 0.03 |
| Statin use | 1.25 | 0.60 | 1.90 | <0.001* | 0.09 |
| Anticoagulant | 1.05 | 0.57 | 1.52 | <0.001* | 0.11 |
| CEAP C class (0–6) | 0.27 | 0.14 | 0.40 | <0.001* | 0.10 |
| CEAP 2–3^1^ | 0.54 | -0.32 | 1.40 | 0.21 | 0.04 |
| CEAP 4–6^1^ | 0.97 | 0.16 | 1.78 | 0.020 | 0.04 |

^1^For this analysis, CEAP class was treated as categorical variable

* Significant at p<0.05 level
